# Descriptions of self-treatment for the middle-aged and elderly in Shanxi, China

**DOI:** 10.1371/journal.pone.0198554

**Published:** 2018-06-11

**Authors:** Rui Wang, Chenjin Ma, Kun Jiang, Ming Li, Shuangge Ma

**Affiliations:** 1 College of Mathematics, Taiyuan University of Technology, Taiyuan, Shanxi, China; 2 School of Statistics, Renmin University of China, Beijing, China; 3 School of Public Health, Yale University, New Haven, Connecticut, United States of America; 4 College of Data Science, Taiyuan University of Technology, Taiyuan, Shanxi, China; Brown University, UNITED STATES

## Abstract

**Objectives:**

Self-treatment is a widespread practice among patients with common symptoms and ailments; it is necessary to explore multiple aspects of it. Notably, there is little research into self-treatment among middle-aged and elderly people, who are more likely to fall ill. Our goals are to provide a comprehensive description of self-treatment and explore associated factors with insurance utilization and expenditures among the middle-aged and elderly populations in China.

**Methods:**

A survey was conducted in July 2016 in Shanxi, China. A stratified sampling scheme was applied to achieve representativeness. A total of 972 subjects were surveyed. Descriptive statistics, t- and Chi-squared tests, multivariate logistic regression, and multivariate linear regression were utilized.

**Results:**

In our study, 772 (79.4%) of the surveyed subjects self-treated during the previous twelve months. Among them, 253 (32.8%) used health insurance. Subjects’ characteristics were associated with insurance utilization and expenditures for self-treatment. Total cost was positively associated with insurance utilization. The subjects with a junior high education (*p*-value < 0.001, aOR = 0.049) and senior high education (*p*-value = 0.020, aOR = 0.146) had a lower probability of using insurance. For both total costs and out-of-pocket costs, subjects who were 51 to 60 years old had lower costs. The subjects who were seriously sick and had a primary school education, as well as enterprise occupations, had higher costs. Self-treatment times were also positively associated with costs. Finally, it was found that subjects who didn’t use insurance had lower total costs.

**Conclusions:**

The prevalence of self-treatment was high (79.4%). Some characteristics were associated with insurance utilization and expenditures in self-treatment. Our results may be helpful for policy interventions, which are needed to further improve the effectiveness of health insurance in China.

## Introduction

In this paper, self-treatment refers to the scenario in which a person uses nonprescription drugs or other approaches to cope with illness conditions [1], covering a wide range of diseases, from minor, common illnesses such as the cold and headaches to complex, chronic diseases [2]. When ill, it is a worldwide practice for individuals to alleviate symptoms through self-treatment [3], especially for the middle-aged and elderly experiencing common symptoms and ailments, such as headaches, colds, and cough [1,4]. Stevenson has showed that many symptoms have been self-treated [5,6]. Self-medication (the use of over-the-counter [OTC] medicines without professional supervision) is the most pervasive form of self-treatment [7]. Traditional therapy is another important form, including massage, acupuncture, skin scraping, and other home remedies [4]. In recent years, self-treatment has become an increasingly prevalent trend, especially in developing countries and regions [8] like Asia. As shown in previous studies, the prevalence of self-treatment varies in different countries, from 9% in the United Kingdom and 13% in the United States to 40–60% in Vietnam, 32% in China, and 71% in India [9,10]. In Asia, self-treatment is a common behavior, and the population utilizing it is huge [1]. However, there has not, to date, been much research on self-treatment. Insurance is a significant factor affecting healthcare behaviors. In China, the current insurance system is composed of basic and commercial insurance. Basic health insurance consists of three schemes for different populations: Urban Employee Basic Medical Insurance, for all urban employers and employees; Urban Resident Basic Medical Insurance, for urban residents as well as children; and New Rural Cooperative Medical Insurance, for rural residents. This basic medical insurance system is conducted by the government and has covered 95% of the total population [11], with the aims of alleviating the economic burden caused by diseases and improving the well-being of individuals. The basic medical insurance can cover most of the costs of hospital-based treatment, but self-treatment is not well-covered. Meanwhile, the supplementary commercial insurance targeted at the high-income group for catastrophic diseases has been developed in recent years, but the coverage is still low in China. Employees usually retire when they are 60 and draw pensions if they have participated in the basic pension insurance program. Therefore, for retirees, the basic medical insurance and pensions are the major sources of health care financing and payment.

Recently, Self-treatment has been integrated into health care systems successfully in many countries [12]. Compared with other more professional hospital-based healthcare approaches (inpatient and outpatient treatments), self-treatment has some advantages to individuals of convenience, speed, accessibility, and lower medical costs [4], complementing the health care system and relieving the burden of limited medical services [7]. More importantly, appropriate self-treatment avoids unnecessary medical treatments and specific medical consultations, resulting in positive effects for both patients and medical health systems [13]. For example, a range of minor symptoms could be alleviated by OTC medicines without visiting hospital, and this could contribute to reducing the costs of treatment as well as traveling and advisement time for patients [14,15]. On the other hand, the negative effects of inappropriate self-treatment cannot be ignored either. Inappropriate self-treatment, including incorrect self-diagnosis, abuse of medications, unexpected side effects and interactions with other drugs, can result in harm to the body [8].

In previous studies, some efforts were made toward investigating the status, patterns, and related factors of self-treatment. A cross sectional study observed a significantly higher proportion of self-treatment in the urban areas of China and identified economic and some individual factors positively associated with the probability of self-treatment [7]. It was discovered by Quynh that 47.5% of subjects who were workers aged 15 to 60 had self-treatment and the presence of acute health symptoms and chronic diseases were significant factors related to the choice of self-treatment in the suburban area of Vietnam [16]. A questionnaire-based study reported that the most common impetuses for self-treatment were to relieve the symptoms of headache; cough, cold, and sore throat; stomachache and fever among college students in Bahrain [17]. In recent years, medical expenditures [18–22] and health insurance [21,23–26], which are closely linked in patients’ vital interests, together with associated factors, have been important topics in many studies. In 2012, a survey which was conducted in two villages in Beijing observed a positive association between out-of-pocket (OOP) medical expenditures and age [19]. Other studies on healthcare expenditure showed that household income, the presence of chronic disease [20] and the usage of insurance [21] [23] had significant influence on healthcare expenditure.

Our study differs from the existing literature in the following ways. First, we explored self-treatment among the middle-aged and elderly populations, who are generally considered to be a relatively unhealthy and financially disadvantaged group in China, inclined to choose self-treatment when ill. Not much research on self-treatment has focused on these populations. Second, this study was conducted in the province of Shanxi, which is representative of other highly populated, agriculture-dominated, underdeveloped areas in China. Shanxi, for example, is a less-developed province, with a per capita gross domestic product [GDP] of 34,919 (RMB) in 2015, compared to the national per capita GDP of 49,992 (RMB) [27]. With the low economic status, health care in such areas is more challenging and may warrant more attention. It is noted that a large area in middle and western China has similar economic and health care conditions as Shanxi. As such, the observations made in Shanxi are expected to hold broadly. It is also necessary to explore multiple aspects of self-treatment in Shanxi. Thus, third, instead of researching one aspect of self-treatment, our study provides a descriptive overview of the topic. This study served three purposes: to detail the characteristics of subjects who employed self-treatments and describe participants’ self-treatment behaviors; to describe the insurance utilization conditions and explore its related factors; to analyses expenditures in self-treatment and examine its associated factors.

## Methods

### Study site

**Fig 1:**
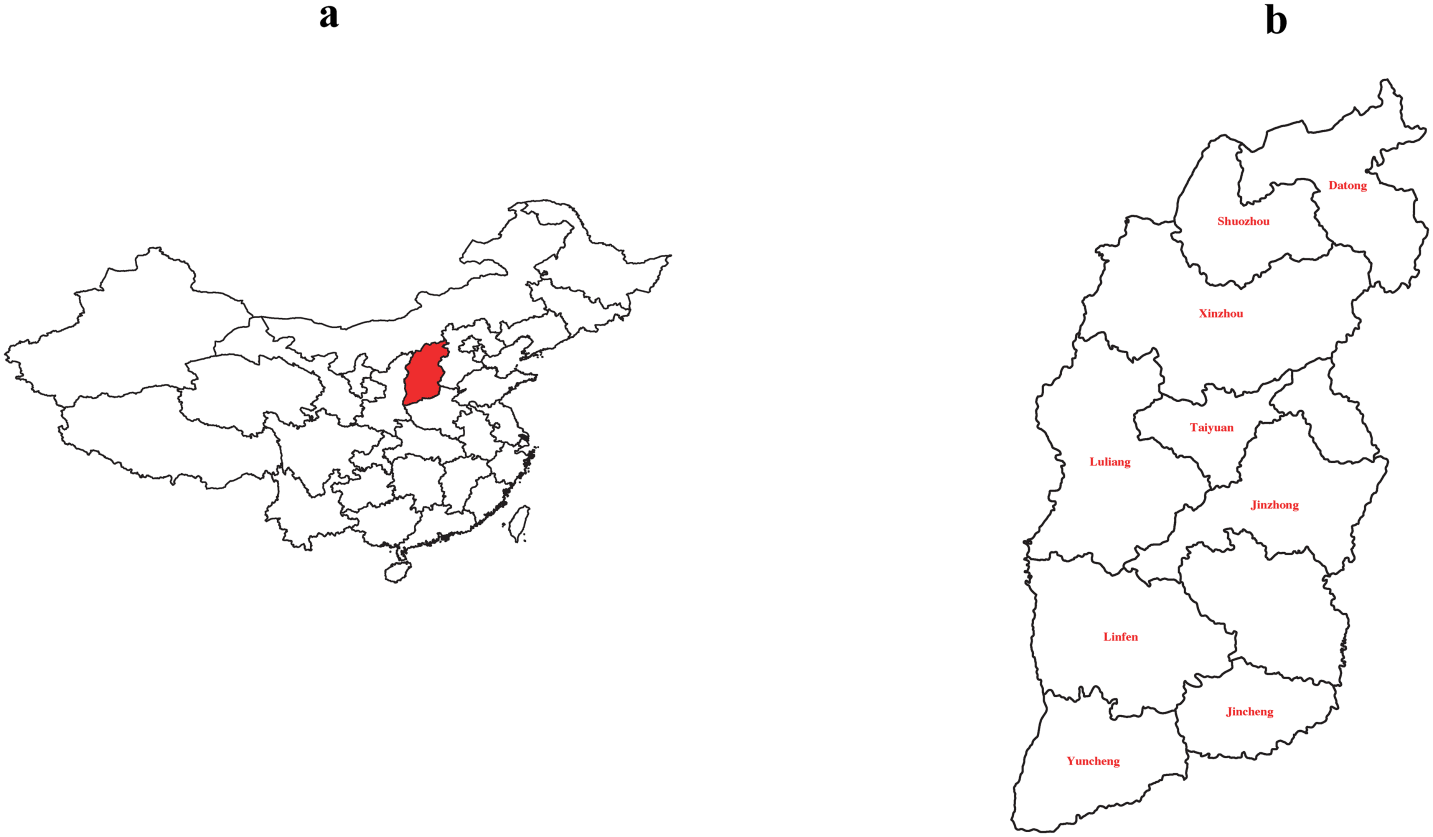
Survey locations map. Note: a. Map of China. The red area is Shanxi Province; b. Map of Shanxi Province. Taiyuan, Shuozhou, Jincheng, Jinzhong, Datong, Linfen, Lvliang, Yuncheng, and Xinzhou are presented. R V3.3.3 software, “maptools” and “ggplot2” packages were used to create this map. The relevant geographic data was downloaded from National Geomatics Center of China http://ngcc.cn/article//sjcg/mndxt/ freely.

A household survey study was conducted in Shanxi in July of 2016. To obtain observations reflecting the actual situation of the whole province, we selected nine cities in Shanxi: three cities with a per capita GDP higher than the average level (Taiyuan, Shuozhou, Jincheng), three around average (Jinzhong, Datong, Linfen), and three lower than average (Lvliang, Yuncheng, Xinzhou). The survey locations are presented in Fig 1.

### Human subject protection

The study was approved by a research ethics review committee at Taiyuan University of Technology. All data were kept strictly confidential. To guarantee the high quality of data collected, all interviewers who were undergraduates from Taiyuan University of Technology were asked to attend training sessions before the survey process began.

### Sampling and recruitment

The surveys were distributed and collected in person. Sampling was conducted in three stages. First, rural and urban communities that are geographically well separated were selected. Macro data such as per capita GDP and population density were considered to achieve representativeness. Within communities, households were randomly selected. Finally, subject was randomly selected in a household. The subjects were those who were older than 45 years and had had at least one disease episode during the previous twelve months. At the beginning of each survey, the interviewer introduced the nature and purpose of the survey. Each interviewee was asked to sign an informed consent form. An interviewee was excluded if he/she refused to participate. Also, to ensure the diversity of samples, only one subject at most from a household was interviewed.

### Study instrument and measures

The questionnaire was composed of two sections: subjects’ characteristics and self-treatment. The sections on subjects’ characteristics inquired about gender, age, marital status, area, education, occupation, type of hukou (which is a household registration issued by the central government and determined urban or rural area of a subject), household/personal capital income per year, household permanent resident population, physical conditions, presence of chronic diseases, and health insurance coverage. The section on self-treatment covered the reasons for self-treating, self-treatment times, self-treated diseases, approaches, insurance utilization for each self-treatment, lost income due to self-treatment, and expenditures for self-treatment. The “total cost” in the study refers to the total cost of treatment and transportation/food/accommodations, as well as lost income during self-treatment. The OOP cost was calculated by subtracting the reimbursement from purchased OTC medicines from the total expense.

### Data collection procedures

A total of 1,100 questionnaires were distributed during the study period, and 972 completed responses were analyzed after a review, yielding a response rate of 88.4%. 200 subjects (20.6%) were found to not have engaged in self-treatment during the previous twelve months. We deleted these data points, so the research population in this study was 772 individuals who engaged in self-treatment during the previous twelve months.

### Data analysis

Due to the consideration of sample diversity, only one person from a household was sampled. Summary statistics were computed on the subjects’ characteristics for the whole cohort, as well as for subgroups stratified by the decision to utilize self-treatment or not. For simplicity, the variable of age was discretized into four groups of different levels. The variables of normal distribution were described by mean and standard deviation, and categorical variables were described by frequency. In the univariate analysis, subjects’ characteristics were compared to different self-treatment statuses to identify factors affecting the decision to self-treat. Additionally, *p*-values were computed from Pearson’s Chi-squared tests for categorical variables and t-tests for continuous variables. The *p*-value of < 0.05 was significant for all tests. Then, multivariate analyses were conducted, accounting for confounding effects. The focus of the first multivariate analysis was insurance utilization, which was a binary response variable. Gender, age, area, marital status, education, occupation, per capita income, health status, the presence of chronic diseases, self-treatment times, total cost, and OOP cost were explanatory variables. A logistic regression analysis was conducted to identify factors associated with insurance utilization, and the adjusted odds ratios (aOR) and *p*-values were computed. In the second analysis, the multivariate linear regression with medical expenditures (total and OOP medical costs) was analyzed. The explanatory variables in the analysis included gender, age, area, marital status, education, occupation, per capita income, health status, the presence of chronic diseases, use of insurance, and self-treatment times. The estimated regression coefficients and their significance levels were computed. All the statistical analyses were conducted using SPSS 17.0.

## Results

### Characteristics of self-treatment

The characteristics of all the participants are presented in Table 1. A total of 772 (79.4%) surveyed subjects received self-treatment during the previous twelve months. Of that number, 537 (55.2%) were male, and 498 (51.2%) were urban residents. In addition, the subjects were mostly distributed in the > 70 age group (33.0%). During the twelve months prior to the survey, 734 (75.5%) subjects had chronic diseases. The probability of using self-treatment was positively associated with chronic diseases (*p*-value = 0.000). Thus, the subjects who had chronic diseases were more likely to administer self-treatment.

**Table 1:** Characteristics of all the participants in the survey.

Characteristics	Self-treatment utilization		*p*-value	Total (n = 972^a^)
	Yes(n = 772^a^)	No(n = 200^a^)		
**Gender**				
Male	420(54.4)	117(58.5)	0.284	537(55.2)
Female	352(45.6)	83(41.5)		435(44.8)
**Age**				
45–50	111(14.4)	41(20.5)	0.155	152(15.6)
51–60	162(21.0)	40(20.0)		202(20.8)
61–70	244(31.6)	53(26.5)		297(30.6)
>70	255(33.0)	66(33.0)		321(33.0)
**Areas**				
Urban	392(50.8)	106(53.0)	0.575	498(51.2)
Rural	380(49.2)	94(47.0)		474(48.8)
**Marital status**				
Single, divorced, widowed	108(14.0)	29(14.5)	0.853	137(14.1)
Married	664(86.0)	171(85.5)		835(85.9)
**Education**				
No schooling	106(13.7)	20(10.0)	0.736	126(13.0)
Primary	213(27.6)	57(28.5)		270(27.8)
Junior high	233(30.2)	64(32.0)		297(30.6)
Senior high	132(17.1)	36(18.0)		168(17.3)
Junior college and more	88(11.4)	23(11.5)		111(11.4)
**Occupation**				
Government	69(8.9)	22(11.0)	0.153	91(9.4)
Enterprises	32(4.1)	17(8.5)		49(5.0)
Farmers	196(25.4)	38(19.0)		234(24.1)
Small private business	46(6.0)	12(6.0)		58(6.0)
Others	43(5.6)	11(5.5)		54(5.6)
Retired	242(31.3)	63(31.5)		305(31.4)
Unemployed	144(18.7)	37(18.5)		181(18.6)
**Per capita income (RMB, mean ±sd)**	23606.49±29913.44	38427.85±190150.12	0.283	26643.52±90140.42
**Health Status**				
Healthy	126(16.3)	34(17.0)	0.796	160(16.5)
General healthy	379(49.1)	91(45.5)		470(48.4)
General unhealthy	177(22.9)	46(23.0)		223(22.9)
Unhealthy	75(9.7)	25(12.5)		100(10.3)
Very unhealthy	15(1.9)	4(2.0)		19(2.0)
**Have chronic diseases**				
Yes	607(78.7)	127(63.5)	0.000	734(75.6)
No	164(21.3)	73(36.5)		237(24.4)
**Total cost (RMB)**	2450.2±4661.8	-		-
**OOP cost (RMB)**	2190.3±4822.2	-		-

^a^ Total may not add up to 200, 772 or 972 due to missing data.

**Fig 2:**
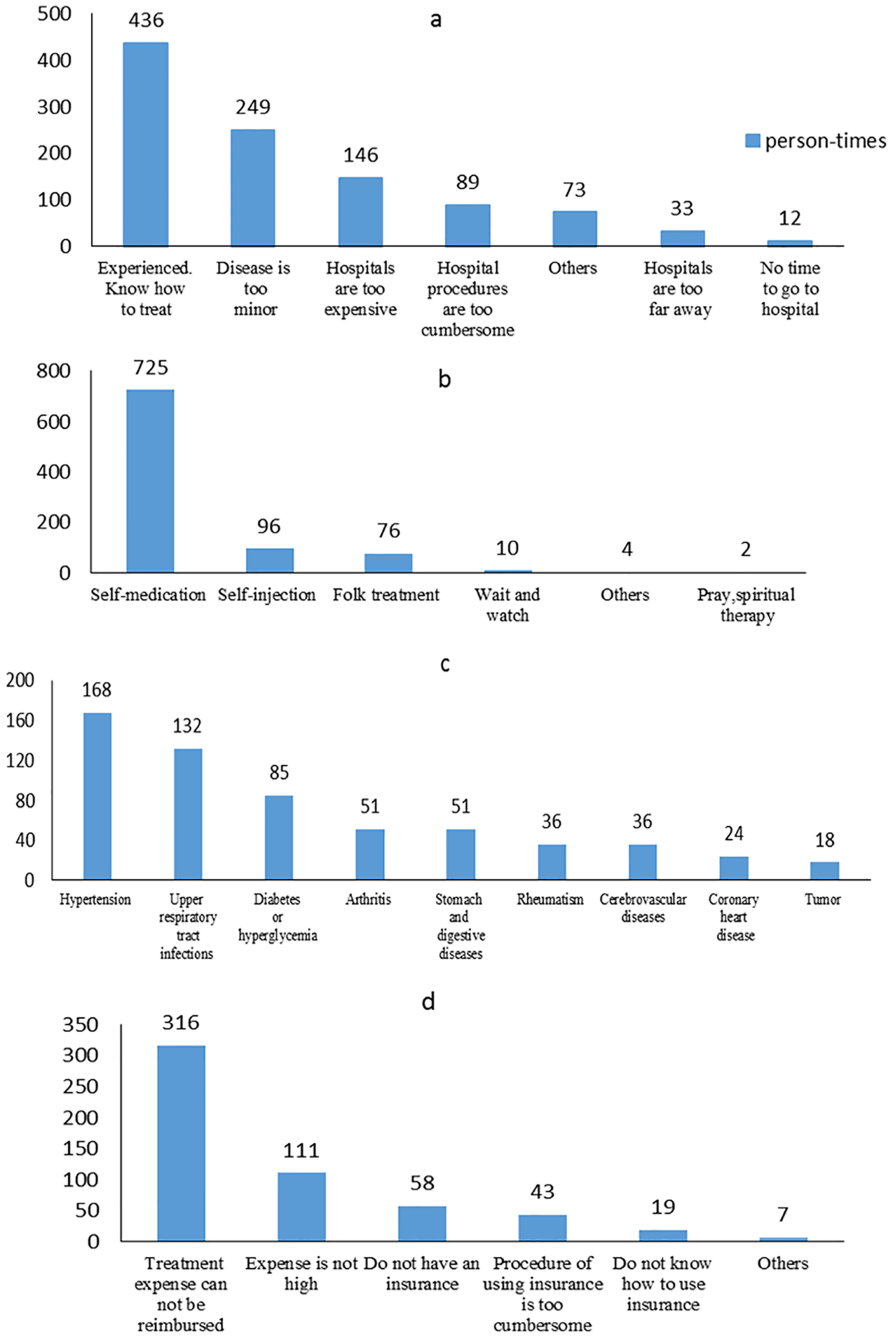
Distributions of self-treatment. Note: (a) self-treatment reasons. (b) self-treatment approaches. (c) self-treated diseases. (d): The reasons for not using insurance in self-treatment.

The reasons for choosing self-treatment are presented in Fig 2a. In total, 436 subjects (56.5%) chose self-treatment due to being experienced at dealing with illness conditions, while 249 subjects thought their disease was too insignificant to go to a hospital. The reasons “hospitals are too expensive” and “hospital procedures are too cumbersome” were chosen by 146 (18.9%) and 89 (11.5%) subjects, respectively. In contrast, the other reasons named above had less than 40 counts.

The approaches used for self-treatment are shown in Fig 2b. Among the 772 subjects who utilized self-treatment, 725 (93.9%) participated in self-medication. As published studies have shown [1,4,13,28], self-medication is the most common form of self-treatment. Besides this, self-injection and folk treatment had 96 and 76 counts, respectively, followed by a “waiting and watching” approach (10 counts). In self-treatment, the most commonly self-treated chronic disease was observed to be hypertension (168 counts). Next came an upper respiratory tract infection (132 counts), followed by diabetes or hyperglycemia (85 counts). Arthritis, stomach, and digestive diseases had 51 counts. Other diseases accounted for less than 5% of the subjects who utilized self-treatment. Some of these results are shown in Fig 2c.

### Insurance

The reasons for not using health insurance are presented in Fig 2d. In this area, 253 (32.8%) subjects used insurance in self-treatment. The most common reason for not using health insurance was that the expense could not be reimbursed (316 counts), accounting for 40.9%. The reasons of “the expense was not high” or not having insurance were chosen by 111 and 58 subjects, respectively. Also, the “procedure of using insurance is too cumbersome” (48 counts) and “do not know how to use insurance” (19 counts) were chosen by a small number of subjects.

The multivariate logistic regression analyses results for insurance utilization are shown in Table 2. Among them, “per capita income” and “self-treatment times” are quantitative variables, while the rest are qualitative variables. Total cost (*p*-value < 0.001, aOR = 1.043) was significantly associated with insurance utilization status. Subjects with higher total costs were more likely to use insurance in self-treatment. Subjects with a junior high education (*p*-value < 0.001, aOR = 0.049) and those with a senior high education (*p*-value = 0.020, aOR = 0.146) were less likely to use insurance in self-treatment.

**Table 2:** Logistic regression model for characteristics and insurance utilization (n = 772).

Characteristics	Estimate	*p*-value	aOR
**Gender (baseline: Male)**			
Female	-0.078	0.870	0.925
**Age (baseline:45–50)**			
51–60	-0.507	0.595	0.602
61–70	-0.886	0.356	0.412
>70	0.700	0.446	2.014
**Areas (baseline: Urban)**			
Rural areas	-0.721	0.294	0.486
**Marital status (baseline: Single, Divorced, Widowed)**			
Married	-0.109	0.844	0.897
**Education (baseline: No schooling)**			
Primary	-0.946	0.099	0.388
Junior high	-3.023	0.000	0.049
Senior high	-1.926	0.020	0.146
Junior college and more	-0.678	0.439	0.508
**Occupation (baseline: Government)**			
Enterprises	1.346	0.315	3.841
Farmers	0.306	0.778	1.358
Small private business	0.892	0.507	2.440
Others	-1.471	0.510	0.230
Retired	1.286	0.173	3.618
Unemployed	0.186	0.857	1.204
**Per capita income (RMB)**	-0.005	0.072	0.995
**Health Status (baseline: Healthy)**			
Just so-so	-0.043	0.943	0.958
Slightly sick	-0.323	0.657	0.724
Sick	0.278	0.771	1.321
Seriously sick	0.030	0.786	1.030
**Have chronic diseases (baseline: No)**			
Yes	-0.561	0.259	0.571
**Self-treatment times**	0.109	0.405	1.116
**Total cost (RMB)**	0.042	0.000	1.043
**OOP cost (RMB)**	-0.042	0.000	0.959

### Expenditure

The average total cost for subjects utilizing self-treatment was 2,450.17 (RMB), the average amount paid by insurance was 445.03 (RMB), the average lost income during treatment was 341.09 (RMB), and the average OOP cost was 2,190.30 (RMB).

The multivariate linear regression analyses results are presented in Table 3. In the first analysis set, age, education, occupation, health status, insurance utilization status, and self-treatment times were found to be associated with total costs. For age, based on the 45–50 group, subjects aged 51–60 years old (estimated coefficient = -1787.858, *p*-value = 0.028) and above 70 years old (estimated coefficient = -1805.095, *p*-value = 0.037) were more likely to have less total costs at a significance level. With the no-schooling group as the baseline, the subjects with a “primary education” were positively associated with total cost at a borderline significance level (estimated coefficient = 1579.521, *p*-value = 0.040). With the government group as the baseline, enterprise employees were observed to have significantly higher total costs (estimated coefficient = 3120.558, *p*-value = 0.025). Subjects with seriously sick status were more likely to have higher total costs. In addition, insurance utilization (estimated coefficient = 1173.302, *p*-value = 0.021) and self-treatment times (estimated coefficient = 633.662, *p*-value < 0.001) were found to be positively related with total cost at a significance level. In the analysis of OOP cost, the factors of age, education, occupation, health status, and self-treatment times were significantly related with OOP cost in self-treatment (*p*-values: 0.037, 0.030, 0.029, 0.050, and < 0.001, respectively), whereas insurance utilization was not a significant factor anymore.

**Table 3:** Multivariate linear regression of expenditure (in RMB).

Characteristics	Total cost of self-treatment(n = 772)		OOP cost of self-treatment(n = 253)	
	Estimate	*p*-value	Estimate	*p*-value
**Gender (baseline: Male)**				
Female	169.323	0.912	275.196	0.555
**Age (baseline:45–50)**				
51–60	-1787.858	0.028	-1620.116	0.037
61–70	-891.750	0.284	-847.992	0.282
>70	-1805.095	0.037	-1372.616	0.095
**Areas (baseline: Urban)**				
Rural	41.618	0.951	124.120	0.846
**Marital status (baseline: Single, Divorced, Widowed)**				
Married	15.336	0.983	-40.079	0.952
**Education (baseline: no schooling)**				
Primary	1579.521	0.040	1598.901	0.030
Junior high	394.478	0.623	276.160	0.720
Senior high	1158.011	0.218	847.642	0.353
Junior college and more	932.904	0.392	786.732	0.460
**Occupation (baseline: Government)**				
Enterprises	3120.558	0.025	2873.304	0.029
Farmers	403.595	0.721	184.420	0.866
Small private business	657.140	0.605	422.071	0.727
Others	-589.131	0.651	-862.391	0.486
Retired	1481.550	0.132	1140.675	0.233
Unemployed	859.901	0.452	327.566	0.766
**Per capita income (RMB)**	-0.004	0.711	-0.004	0.715
**Health status (baseline: Healthy)**				
Just so-so	-800.134	0.235	-1081.132	0.099
Slightly sick	-616.600	0.429	-662.293	0.383
Sick	202.177	0.833	218.703	0.812
Seriously sick	3566.101	0.048	3323.939	0.050
**Have chronic diseases (baseline: No)**				
Yes	538.173	0.372	149.380	0.798
**Use insurance (baseline: No)**				
Yes	1173.302	0.021	153.307	0.756
**Self-treatment times**	633.662	0.000	532.110	0.000

## Discussion

### Characteristics of self-treatment

As an alternative to hospital-based approaches, the role self-treatment plays in health care should not be ignored, as described earlier. In this study, the prevalence (79.4%) of self-treatment in Shanxi was high among the middle-aged and elderly populations. Among the self-treated patients, 93.9% opted for OTC medicines, which was the most popular self-treatment behavior surveyed in previous studies [29,30]. The diseases self-treated were hypertension, diabetes or hyperglycemia, and upper respiratory tract infections, and these diseases are common among the middle-aged and elderly. This point is consistent with the results of previously published studies [1,28]. As discussed above, self-treatment is common and serves as a suitable complementary to hospital-based treatment for treating these common chronic diseases. Clinical doctors should be aware that there are still some patients with chronic diseases who choose self-treatment instead of visiting hospital service. However, the basic health insurance system in China has been experiencing fast developments in the past decade and will continue developing in the next years, with more chronic diseases covered and easier to access, which may cause more chronic diseases treated in hospitals in the next few years.

### Insurance

Most of our results are consistent with previously published studies [23,31]. In the multivariate logistic regression, only education and medical expenditure (total/OOP costs) were found to be significantly associated with insurance utilization, accounting for confounding effects. The effects of health expenses might exacerbate poverty and poor health, particularly for subjects with low incomes [32]. Basic medical insurance is the major source of health care financing and payment in China [33], with the aims of alleviating the economic burden caused by diseases and improving the well-being of individuals. Therefore, the target populations for insurance are economically disadvantaged groups (for example, the uneducated group). It is, therefore, not surprising that compared with the uneducated, those with junior and senior high educations are more likely to self-treat without insurance. As discovered in previous studies [31], health insurance increased the risk of medical spending. And, more total cost usually corresponds to more serious diseases and a higher probability of using insurance. On the other hand, a small amount of reimbursement by insurance could reduce the actual medical costs and corresponds to narrowly less OOP costs. In contrast, age and area were not significant factors.

From the perspective of government, it is expected to carry out universal education on health care, especially for the disadvantaged population. It is easier for subjects with higher socioeconomic statuses to learn about updated insurance policies and related instructions. Enabling health insurance to play an effective role in relieving the pecuniary burden on self-treatment patients is suggested.

### Expenditure

One purpose of our study was to explore the relationship(s) between certain characteristics and expenditures in self-treatment. For the total cost and OOP cost of self-treatment, the multivariate regression results show that subjects 51–60 years old had lower costs. The subjects who were seriously sick and who had primary school educations or enterprise occupations had higher costs. Self-treatment times are positively associated with the cost. Also, it was found that subjects who didn’t use insurance had a lower total cost.

The results above have intuitive and reasonable interpretations. In China, the legal retirement age is no less than 50. In the 45–50 age group, most of the middle-aged subjects were still at work. Since the lost income during treatment was also included in total expenses in this survey, we found that the “youngest” group was likely to incur more lost income and total/OOP medical expenses. The occupational differential is also recognized in China. Small, private enterprise employees are likely to earn more compared to government staff. Moreover, the prevalence of self-treatment increased with higher education levels, representing greater knowledge of medicines and economic power [34]. Subjects with higher educational or occupational levels are more willing to treat themselves frequently and have easier access to those relatively costly traditional therapies that cannot be reimbursed, therefore leading to more total/OOP costs. On the other hand, patients with less education may give up self-treatment because of a lack of knowledge about pharmaceutical products [35], which results in less medical expenditure. What’s more, the “very unhealthy” group and self-treated times all had significantly positive associations with total/OOP costs, with more unhealthy statuses and self-treated times corresponding to more serious diseases and medical costs (total/OOP costs). Still, while using health insurance in self-treatment significantly increased the total cost, it did not make a significant difference in OOP costs. Because insurance reimburses part of the medical expenditure, people who use insurance spend more money for better treatment. In fact, the OOP cost will not change. The reimbursement for OTC medicines narrows the gap but makes no significant difference in OOP costs between insurance utilization statuses. This result reflecting the role health insurance plays in reducing medical expenditures in self-treatment is limited. The findings above were partly consistent with previously published ones [36,37]. Marriage status and per capita income, though, were found to be insignificant. And chronic diseases were also found to be insignificant, which differs from previous studies [38,39]. The reasons leading to these differences may be because the study focused on the population of the middle-aged and elderly and also medical expenditure may vary with local economic growth or alteration of the attitude toward receiving treatment with medicine.

## Limitations

With the survey in Shanxi, the cross-sectional, descriptive data were collected and had some limitations. First, limited resources made the sample size also limited, and the information collected might not contain other important variables. On the other hand, the information collected in this study basically contains all the variables we needed, as well as the variables studied in previous research, and thus could show a comprehensive-enough overview of self-treatment. Second, this study still has some information bias that could not be avoided in such a cross-sectional survey, including potential recall bias, especially for information on illness conditions and medical expenditure. However, there is no complete and reliable system or hospital-based database to record information about self-treatment at any time. The only way to collect information about self-treatment is using questionnaires. Third, this was a cross-sectional survey, which did not allow us to assess causality or the directionality of relationships. Fourth, this study didn’t cover all age groups, instead focusing only on middle-aged and elderly subjects (45 years old and older). However, middle-aged and elderly subjects are generally considered to be a relatively unhealthy and financially disadvantaged population in China, inclined to choose self-treatment when ill. Thus, this age group is representative. Finally, as with other studies [1,3,4], we chose 0.05 as significance level, it might be suspect because of potential multiple comparisons. Associations with less facial plausibility should be considered suggestive, but that these results would need to be confirmed.

## Conclusions

This study focused on a comprehensive description of self-treatment among the middle-aged and elderly populations in China. It was found that self-treatment has a high prevalence (79.4%). The results of this study show that self-treatment is common and provide a better understanding of self-treatment behavior. Age and education were found to be associated with insurance utilization, occupation, health status, insurance utilization status, and self-treatment times were associated with total costs, the factors of age, education, occupation, health status, and self-treatment times were significantly related with OOP cost. Such results may be helpful for policy interventions, which are needed to further improve the effectiveness of health insurance among the relatively unhealthy and financially disadvantaged populations in China. Can self-treatment be a good complementary approach to treat some high cost chronic diseases? What are the common healthcare outcomes in relation to self-treatment? These could be future research topics.

## Supporting information

S1 File Questionnaire Chinses version. (DOCX)

S2 File Questionnaire English version. (DOCX)
