# Supplementary material for: Descriptions of self-treatment for the middle-aged and elderly in Shanxi, China
Source: PLoS One. 2018 Jun 11;13(6):e0198554. doi: 10.1371/journal.pone.0198554 (PMC5995374; doi:10.1371/journal.pone.0198554)
Supplement: S1 File — (DOCX) [file pone.0198554.s001.docx]

**山西省医疗消费和养老状况评价**

**跟踪调查问卷**

我是太原理工大学的学生，这是我的介绍信和学生证，正在进行“中国养老和医疗保障政策评价”课题研究。希望通过调查了解老百姓的真实感受，为国家养老和医疗保险政策调整提供建议。为了获得准确的数据，请您依据实际情况，回答访问员提出的问题。如果对您的生活和工作造成不便，我们深表歉意，请您理解和帮助我们的工作。对问卷中问题的回答，您只要根据平时的想法和实际情况回答就行。对于您的回答，我们将按照《统计法》的规定，严格保密，并且只用于统计分析谢谢您的合作。

[问卷处理记录]

（此处不用填写）

访问员（签名）：__________

一审（签名）：_________

二审（签名）：__________

录入人员（签名）：__________

**1. 请问您家里45岁以上常住人口有多少人？【 】人**

**（*如果没有，填0，结束访问*）**

**2. 请问您家里45岁以上常住人口中，过去一年有几人生过病？【 】人**

**（*如果没有，填0，结束访问*）**

**第一部分：个人基本情况**

A1. 您的性别（***访员自填***）【 】

1. 男 2. 女

A2. 您的年龄【 】岁

A3. 您的婚姻状况【 】

1. 单身 2. 已婚 3. 离婚 4. 丧偶 5. 同居 99. 其他

A4. 您的学历是【 】

1. 文盲/半文盲 2.小学 3. 初中 4.高中 5.大专

6. 大学本科及以上 99. 其他

A5. 您的职业是【 】

1. 行政机关及事业单位 2. 企业/有雇主 3. 务农 4. 个体经营者 5. 其他就业者 6. 离退休人员 7. 无业 8. 其他非就业者

A6. 您的户口是【 】

1. 当地非农户口 2. 当地农村户口 3. 外地非农户口 4. 外地农村户口

B5. 在过去的一年中，请问您**个人的总收入**（*实物收入请折算为现金收入，定义见附录*）是多少？

| **百万** | **十万** | **万位** | **千位** |  |
| --- | --- | --- | --- | --- |
| __ | __ | __ | __ | 元 |

**第二部分：家庭成员医疗消费情况**

B1. 请问您是否有医疗保险？【】

1. 有 2. 没有

B2. 请问您的健康状况如何？【 】

1. 健康 2. 一般 3. 比较不健康 4. 不健康 5. 非常不健康

B3.您是否有慢性病？

0. 没有***（跳至D6）*** 1. 有

**自我治疗情况：**

B4. 过去一年您/是否有过自我治疗？【 】

（*自我治疗的定义：没有经过医院诊断的，以治病为目的的买药、按摩、刮痧等行为*）

1. 是 2. 否（***跳至E1***）

B5.过去一年您一共自我治疗了多少次？【】

| **问题** | **自我治疗次数** | | | | | |
| --- | --- | --- | --- | --- | --- | --- |
|  | **1** | **2** | **3** | **4** | **5** | **6** |
| B6. 您**每次**是因为什么病进行自我治疗的？  1.上呼吸道感染2. 关节炎 3.风湿病 4. 糖尿病或血糖高 5. 高血压  6. 脑血管疾病7. 慢性风湿性心脏病8. 缺血性心脏病9. 冠心病  10. 中风11. 肺心病12. 脑卒中13. 肿瘤 14. 慢性阻塞性肺部疾患（COPD）  15. 哮喘 16. 慢性肝病和肝硬化17. 胃部疾病或消化系统疾病  18. 肾脏疾病 19. 老年痴呆20. 帕金森氏综合症 21. 精神类疾病  22. 传染性疾病23.泌尿性疾病24.生殖系统疾病 25. 皮肤疾病 26.颈椎病、骨质疏松症等27. 损伤28. 其他（请注明____________________） | 【】 | 【】 | 【】 | 【】 | 【】 | 【】 |
| B7. 您都用了哪些自我治疗的方式（***可多选***）？  1. 自己找药/买药 2自己打针/打点滴 3民间方法治疗（如刮痧、针灸等）  4.去求神拜佛或做法事（*定义见附录*） 5. 不采取任何措施，等病慢慢好  99. 其他（请注明_______） | 【】 | 【】 | 【】 | 【】 | 【】 | 【】 |
| B8. 您生病不去医院看病的最主要原因是什么（***可多选***）？  1. 医疗费用太贵 2. 离医院或其它医疗机构太远 3. 医院的手续太麻烦  4.挂不上号 5. 不相信医生 6. 医生态度不好  7.觉得是小病，没必要去医院 8.有经验了，可以自己治 9. 无人陪同去医院  10.医院没办法治 11. 没时间去医院 99.其他（请注明_______） | 【】 | 【】 | 【】 | 【】 | 【】 | 【】 |

B9. 过去一年您自我治疗一共花费多少钱？（包括治疗的费用、交通、住宿等等的费用）

99999 拒答

99998 不知道

| **十万** | **万位** | **千位** | **百位** | **十位** |  |
| --- | --- | --- | --- | --- | --- |
| __ | __ | __ | __ | __ | 元 |

B10. 在支付他/她的自我治疗总费用时，是否使用了保险？【】

1. 是（***跳至B10.2***） 2. 否（***跳至B10.1***）

B10.1没使用保险的最主要原因是什么（可多选）？

1. 没有保险

2. 因为使用保险的流程过于复杂

3. 因为费用不高

4. 因为治疗费用不包含在使用保险的范围里

5. 因为不知如何使用保险

99. 其他（请注明）

B10.2在过去一年的自我治疗总费用中，保险支付了多少钱？

| **十万** | **万位** | **千位** | **百位** | **十位** |  |
| --- | --- | --- | --- | --- | --- |
| __ | __ | __ | __ | __ | 元 |
