# Supplementary material for: Descriptions of self-treatment for the middle-aged and elderly in Shanxi, China
Source: PLoS One. 2018 Jun 11;13(6):e0198554. doi: 10.1371/journal.pone.0198554 (PMC5995374; doi:10.1371/journal.pone.0198554)
Supplement: S2 File — (DOCX) [file pone.0198554.s002.docx]

No.______

Self-treatment for the middle-aged and elderly in Shanxi, China

Hello! I am an interviewer at the Taiyuan University of Technology. A research project on " China Survey on Pension and Healthcare " are conducting. The investigation process may delay you a few minutes, the data obtained only as a sample data analysis, we are committed not to disclose your personal information, hoping to get your cooperation. Thank you for your support!

[Questionnaire processing record]

(Do not need to fill here)

Interviewer (signed): __________

First instance (signature): _________

Second instance (signature): __________

Enter (Signature): __________

1. How many people who was 45 years old and above in your household?

(***If not, fill 0, and end visit***)

2. How many people who was 45 years old and above have been sick during the past 12 months in your household?

(***If not, fill 0, and end visit***)

**Part one: Basic information**

A1. Your gender: _____

A2. Your age: _____

A3. Your marital status: _____

1. Single 2. Married 3. Divorced 4. Widowed

5.Living together 99. Others

A4. Your education: _____

1.No schooling 2. Primary 3. Junior high 4. Senior high

5. Junior college 6. College and above 99. Others

A5. Your occupation: _____

1.Government 2. Enterprises 3. Farmers 4. Small private business

5.Others 6. Retired 7. Unemployed 8. Other non-employed.

A6. Your hukou: _____

1. Local non-agricultural hukou 2. Local agricultural hukou

3. Foreign non-agricultural hukou 4. Foreign agricultural hukou

A7. What is your total personal income in the past year?

| 1,000,000 | 100,000 | 10,000 | 1,000 |  |
| --- | --- | --- | --- | --- |
|  |  |  |  | RMB |

**Part two: Self-treatment information**

B1. Do you have medical insurances?

1. Yes 2. No

B2. What is the status of your health (including physiological, psychological and social aspects)?

1. Healthy status 2. General healthy status 3. General unhealthy status

4. Unhealthy status 5. Very unhealthy status

B3. Do you have any chronic diseases?

0. No 1. Yes

The following questions focus on the past 12 months.

B4. Have you ever had self-treatment?

(The definition of self-treatment: The action of buying medicine, taking massage, scraping and others for the purpose of curing without hospital professional diagnosis)

1. Yes 2. No ***(finish here)***

B5. How many times have you ever self-treated? ________

| **Questions** | **Times of self-treatment** | | | | | |
| --- | --- | --- | --- | --- | --- | --- |
|  | **1** | **2** | **3** | **4** | **5** | **6** |
| B6. What kind of disease did you get self-treated for each time?  1. Upper respiratory tract infection 2. Arthritis; 3. Rheumatism 4. Diabetes; 5. Hypertension; 6. Cerebrovascular disease; 7. Chronic rheumatic heart disease; 8. Ischemic heart disease; 9. Coronary heart disease; 10. Stroke; 11. Pulmonary heart disease; 12. Brain stroke; 13. Tumors; 14. Chronic Obstructive Pulmonary Disease(COPD); 15. Asthma; 16. Chronic liver disease and cirrhosis; 17. Stomach disease or digestive system diseases; 18. Kidney disease; 19. Alzheimer’s disease; 20. Parkinson’s disease; 21. Mental illness; 22. Infectious Diseases; 23. Urological diseases; 24. Reproductive system diseases; 25. Skin diseases; 26. Cervical spondylosis, osteoporosis and so on; 27. Injury; 28. Others (please specify___________) | 【】 | 【】 | 【】 | 【】 | 【】 | 【】 |
| B7. Which self-treatment approaches did you use?  1. Self-medication 2. Self-medication 3. Folk treatment (such as scarping, acupuncture, etc.) 4. Pray, spiritual therapy 5. Wait and Watch  99. Others (please specify________) | 【】 | 【】 | 【】 | 【】 | 【】 | 【】 |
| B8. The reason why you didn’t go to hospital:  1. Too expensive 2. Too far away 3. Too cumbersome 4. Unable to register in hospitals 5. Do not trust doctors 6. Doctors’ attitude is not good 7. Disease is too minor 8. Experienced. Know how to treat 9. No one accompany to the hospital 10. Doctors in hospitals are unable to cure the disease. 11. No time to go to hospital | 【】 | 【】 | 【】 | 【】 | 【】 | 【】 |

B9. How much have you spent on self-treatment, including the treatment, transportation, accommodation?

| 100,000 | 10,000 | 1,000 | 100 | 10 |  |
| --- | --- | --- | --- | --- | --- |
|  |  |  |  |  | RMB |

B10. Has the health insurance been used to cover the cost in your self-treatment?

1. Yes **(*Turn to B10.2*)** 2. No **(*Turn to B10.1*)**

B10.1. What was the main reason for not using insurance in your self-treatment?

1. Do not have an insurance. 2. Procedure of using insurance is too cumbersome. 3. Expense is not high. 4. Treatment expense cannot be reimbursed.

5. Do not know how to use insurance. 99. Others (please specify________)

B10.2 How much has insurance reimbursed out of the total cost in your self-treatment?

| 100,000 | 10,000 | 1,000 | 100 | 10 |  |
| --- | --- | --- | --- | --- | --- |
|  |  |  |  |  | RMB |
